# Hexaaqua­manganese(II) 4,4′-(1,2-dihy­droxy­ethane-1,2-di­yl)dibenzoate monohydrate

**DOI:** 10.1107/S1600536810022300

**Published:** 2010-06-16

**Authors:** Cheng-Jun Hao, Yun-Li Cao

**Affiliations:** a College of Chemistry and Chemical Engineering, Pingdingshan University, Pingdingshan 467000, People’s Republic of China

## Abstract

In the title compound, [Mn(H_2_O)_6_](C_16_H_12_O_6_)·H_2_O, the [Mn(H_2_O)_6_]^2+^ complex cation lies on a mirror plane, the 4,4′-(1,2-dihy­droxy­ethane-1,2-di­yl)dibenzoate anion is located on an inversion center and the solvent water mol­ecule also lies on a mirror plane. Extensive O—H⋯O hydrogen-bonding inter­actions between the cations, anions and water mol­ecules stabilize the three-dimensional network.

## Related literature

For the intriguing architectures and potential applications of polymeric coordination networks, see: Carlucci *et al.* (2003 ▶); Rosi *et al.* (2003 ▶).
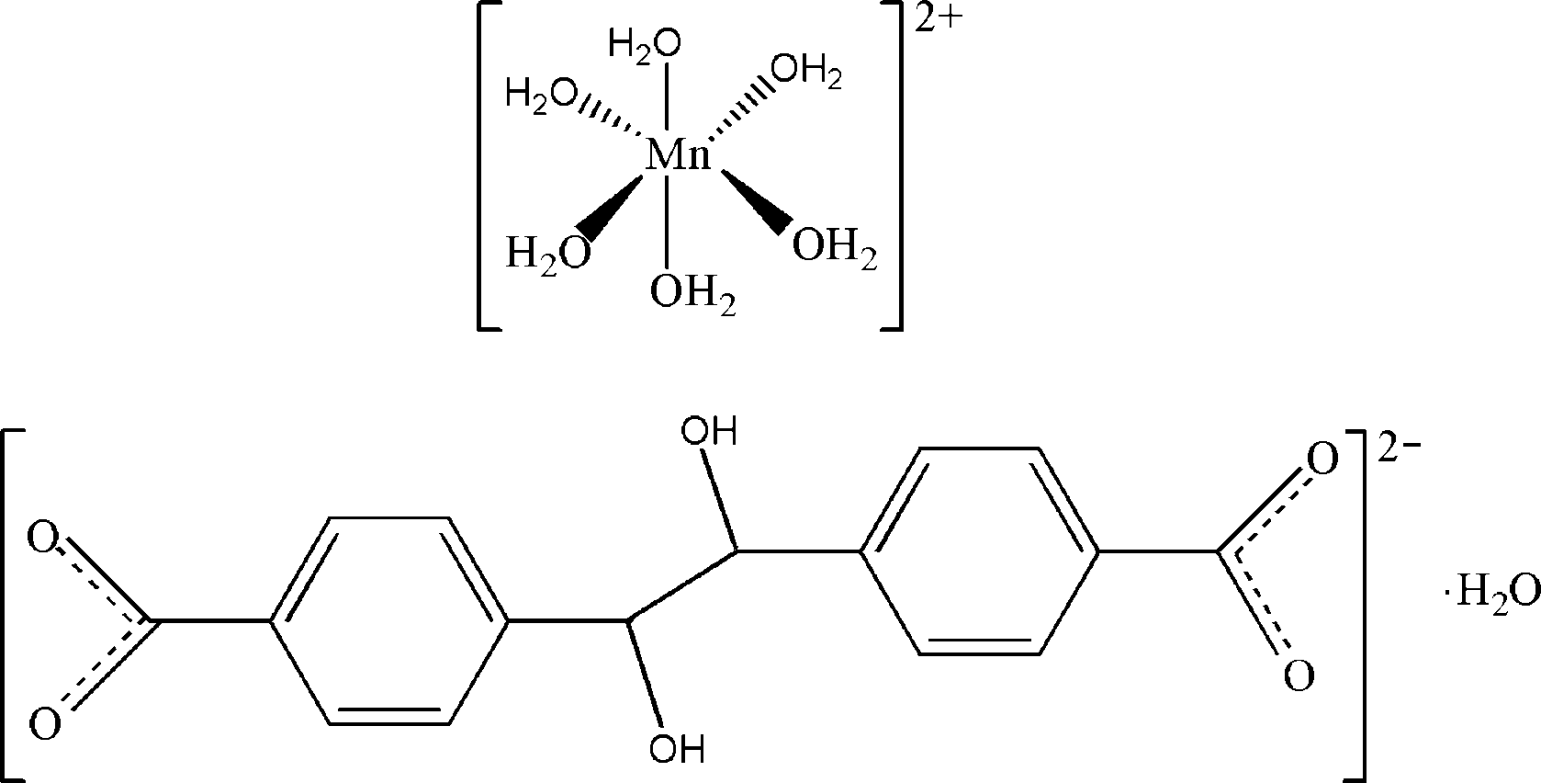


## Experimental

### 

#### Crystal data

[Mn(H_2_O)_6_](C_16_H_12_O_6_)·H_2_O
*M* *_r_* = 481.31
Monoclinic, 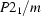

*a* = 6.0803 (6) Å
*b* = 20.643 (2) Å
*c* = 8.6610 (9) Å
β = 104.420 (1)°
*V* = 1052.84 (19) Å^3^
*Z* = 2
Mo *K*α radiation
μ = 0.69 mm^−1^
*T* = 298 K
0.42 × 0.21 × 0.18 mm

#### Data collection

Bruker SMART 1000 CCD diffractometer
Absorption correction: multi-scan (*SADABS*; Bruker, 2001 ▶) *T*_min_ = 0.760, *T*_max_ = 0.886
5275 measured reflections
1899 independent reflections
1647 reflections with *I* > 2σ(*I*)
*R*_int_ = 0.024

#### Refinement

*R*[*F* ^2^ > 2σ(*F* ^2^)] = 0.057
*wR*(*F* ^2^) = 0.140
*S* = 1.23
1899 reflections
142 parameters
H-atom parameters constrained
Δρ_max_ = 0.84 e Å^−3^
Δρ_min_ = −0.33 e Å^−3^

### 

Data collection: *SMART* (Bruker, 2007 ▶); cell refinement: *SAINT* (Bruker, 2007 ▶); data reduction: *SAINT*; program(s) used to solve structure: *SHELXS97* (Sheldrick, 2008 ▶); program(s) used to refine structure: *SHELXL97* (Sheldrick, 2008 ▶); molecular graphics: *SHELXTL* (Sheldrick, 2008 ▶); software used to prepare material for publication: *SHELXTL*.

## Supplementary Material

Crystal structure: contains datablocks I, global. DOI: 10.1107/S1600536810022300/hy2318sup1.cif

Structure factors: contains datablocks I. DOI: 10.1107/S1600536810022300/hy2318Isup2.hkl

Additional supplementary materials:  crystallographic information; 3D view; checkCIF report

## Figures and Tables

**Table 1:** Hydrogen-bond geometry (Å, °)

*D*—H⋯*A*	*D*—H	H⋯*A*	*D*⋯*A*	*D*—H⋯*A*
O3—H3⋯O1^i^	0.82	2.02	2.830 (5)	172
O4—H4*C*⋯O1^ii^	0.85	1.86	2.712 (4)	177
O5—H5*C*⋯O4^iii^	0.85	1.93	2.777 (6)	175
O5—H5*D*⋯O8^iii^	0.85	1.88	2.728 (7)	175
O6—H6*C*⋯O3^iv^	0.85	1.99	2.840 (5)	178
O6—H6*D*⋯O8	0.85	2.19	3.040 (6)	178
O7—H7*C*⋯O1^v^	0.85	1.95	2.799 (5)	180
O7—H7*D*⋯O2^ii^	0.85	1.82	2.673 (4)	180
O8—H8*C*⋯O2^vi^	0.85	1.92	2.767 (5)	172

Symmetry codes: (i) 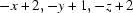; (ii) 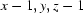; (iii) 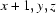; (iv) 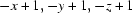; (v) 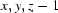; (vi) 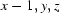.

**Fig. 1.**
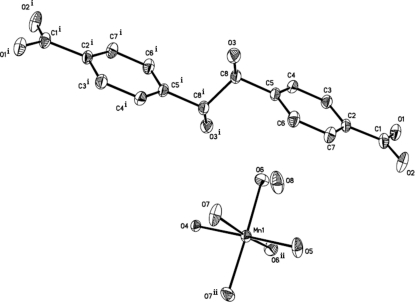
Molecular structure of the title compound. Displacement ellipsoids are shown at the 30% probability level. H atoms and water molecule are omitted for clarity. [Symmetry codes: (i) 1-x, 1-y, 1-z; (ii) x, 3/2-y, z.]

**Fig. 2.**
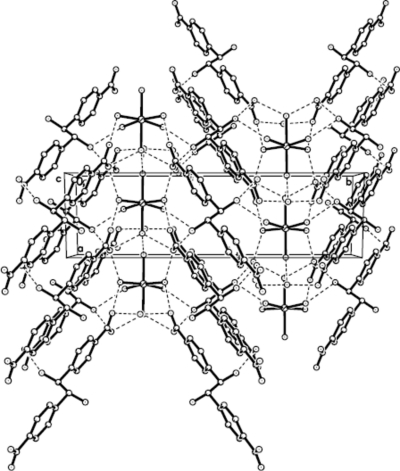
View of the three-dimensional network constructed by O—H···O hydrogen bonds (dashed lines). H atoms are omitted for clarity.

## supplementary crystallographic information

### Comment

Current interest in polymeric coordination networks is rapidly expanding for their intriguing architectures (Carlucci *et al.*, 2003) and potential applications (Rosi *et al.*,  2003). We have reacted 1,2-bis(4-carboxyphenyl)-1,2-ethanediol with MnCl_2_ under hydrothermal conditions to obtain the title compound and its structure is reported here.

As illustrated in Fig. 1, the title compound contains one [Mn(H_2_O)_6_]^2+^ complex cation lying on a mirror plan, one 1,2-dihydroxyethane-1,2-bis(4-benzenecarboxylate) anion located on an inversion center and one solvent water molecule lying on a mirror plan. The carboxylate group lies in the plane of the benzene ring as indicated by the O1—C1—C2—C3 and O2—C1—C2—C7 torsion angles of -3.0 (6) and -1.2 (6)°. The benzene ring is nearly planar with maximum deviations from the mean plane being -0.003 (6) Å for C6. The cation, anion and solvent water molecule interact via O—H···O hydrogen bonds, consolidating the three-dimensional network (Fig. 2, Table 1).

### Experimental

A mixture of MnCl_2_ (0.1 mmol, 0.013 g), 1,2-bis(4-carboxyphenyl)-1,2-ethanediol (0.1 mmol, 0.03 g) and 10 ml of H_2_O was sealed in a 20 ml Telflon-lined stainless steel vessel and heated at 303 K for 2 d. Colorless crystals were obtained when the solution was cooled to room temperature slowly.

### Refinement

H atoms bound to C atoms were placed at calculated positions and were treated as riding on the parent atoms, with C—H = 0.93 (aromatic) and 0.98 (CH) Å and with *U*_iso_(H) = 1.2*U*_eq_(C). H atoms of hydroxyl group and water molecules were located in a difference Fourier map and refined as riding, with O—H = 0.85 Å and *U*_iso_(H) = 1.2(1.5 for hydroxyl)*U*_eq_(O).

### Crystal data

**Table:**

[Mn(H_2_O)_6_](C_16_H_12_O_6_)·H_2_O	*F*(000) = 502
*M**_r_* = 481.31	*D*_x_ = 1.518 Mg m^−^^3^
Monoclinic, *P*2_1_/*m*	Mo *K*α radiation, λ = 0.71073 Å
Hall symbol: -P 2yb	Cell parameters from 2215 reflections
*a* = 6.0803 (6) Å	θ = 2.5–24.0°
*b* = 20.643 (2) Å	µ = 0.69 mm^−^^1^
*c* = 8.6610 (9) Å	*T* = 298 K
β = 104.420 (1)°	Block, colorless
*V* = 1052.84 (19) Å^3^	0.42 × 0.21 × 0.18 mm
*Z* = 2	

### Data collection

**Table:**

Bruker SMART 1000 CCD diffractometer	1899 independent reflections
Radiation source: fine-focus sealed tube	1647 reflections with *I* > 2σ(*I*)
graphite	*R*_int_ = 0.024
φ and ω scans	θ_max_ = 25.0°, θ_min_ = 2.0°
Absorption correction: multi-scan (*SADABS*; Bruker, 2001)	*h* = −7→6
*T*_min_ = 0.760, *T*_max_ = 0.886	*k* = −24→22
5275 measured reflections	*l* = −10→9

### Refinement

**Table:**

Refinement on *F*^2^	Primary atom site location: structure-invariant direct methods
Least-squares matrix: full	Secondary atom site location: difference Fourier map
*R*[*F*^2^ > 2σ(*F*^2^)] = 0.057	Hydrogen site location: inferred from neighbouring sites
*wR*(*F*^2^) = 0.140	H-atom parameters constrained
*S* = 1.23	*w* = 1/[σ^2^(*F*_o_^2^) + (0.0292*P*)^2^ + 3.0592*P*] where *P* = (*F*_o_^2^ + 2*F*_c_^2^)/3
1899 reflections	(Δ/σ)_max_ < 0.001
142 parameters	Δρ_max_ = 0.84 e Å^−^^3^
0 restraints	Δρ_min_ = −0.33 e Å^−^^3^

### Fractional atomic coordinates and isotropic or equivalent isotropic displacement parameters (Å^2^)

**Table:**

	*x*	*y*	*z*	*U*_iso_*/*U*_eq_	
Mn1	0.65486 (15)	0.7500	0.46088 (10)	0.0289 (3)	
O1	1.0818 (5)	0.64065 (16)	1.2067 (4)	0.0444 (8)	
O2	1.3265 (5)	0.64967 (19)	1.0567 (4)	0.0567 (10)	
O3	0.6710 (6)	0.42818 (15)	0.5229 (4)	0.0472 (9)	
H3	0.7538	0.4103	0.6004	0.071*	
O4	0.2836 (6)	0.7500	0.3483 (5)	0.0296 (9)	
H4C	0.2168	0.7166	0.3015	0.036*	
O5	1.0130 (7)	0.7500	0.5635 (5)	0.0533 (14)	
H5C	1.0885	0.7500	0.4931	0.064*	
H5D	1.1062	0.7500	0.6548	0.064*	
O6	0.5811 (6)	0.67978 (16)	0.6304 (4)	0.0477 (8)	
H6C	0.5029	0.6476	0.5861	0.057*	
H6D	0.5095	0.6985	0.6904	0.057*	
O7	0.6863 (5)	0.67266 (18)	0.2996 (4)	0.0548 (10)	
H7C	0.8061	0.6629	0.2710	0.066*	
H7D	0.5724	0.6653	0.2220	0.066*	
O8	0.3346 (10)	0.7500	0.8464 (6)	0.090 (2)	
H8C	0.3323	0.7167	0.9035	0.109*	
C1	1.1422 (7)	0.6306 (2)	1.0792 (5)	0.0377 (11)	
C2	0.9858 (7)	0.5932 (2)	0.9471 (5)	0.0321 (10)	
C3	0.7845 (7)	0.5675 (2)	0.9661 (5)	0.0364 (10)	
H3A	0.7415	0.5747	1.0605	0.044*	
C4	0.6454 (7)	0.5311 (2)	0.8455 (5)	0.0371 (10)	
H4	0.5098	0.5144	0.8597	0.045*	
C5	0.7062 (7)	0.5195 (2)	0.7053 (5)	0.0335 (10)	
C6	0.9078 (8)	0.5454 (2)	0.6850 (5)	0.0398 (11)	
H6	0.9505	0.5381	0.5906	0.048*	
C7	1.0457 (7)	0.5820 (2)	0.8053 (5)	0.0387 (11)	
H7	1.1802	0.5993	0.7905	0.046*	
C8	0.5518 (8)	0.4795 (2)	0.5750 (5)	0.0365 (10)	
H8	0.4286	0.4613	0.6160	0.044*	

### Atomic displacement parameters (Å^2^)

**Table:**

	*U*^11^	*U*^22^	*U*^33^	*U*^12^	*U*^13^	*U*^23^
Mn1	0.0245 (5)	0.0337 (5)	0.0272 (5)	0.000	0.0039 (4)	0.000
O1	0.0364 (17)	0.051 (2)	0.0399 (18)	0.0007 (15)	−0.0016 (14)	−0.0157 (15)
O2	0.0333 (19)	0.078 (3)	0.052 (2)	−0.0144 (18)	−0.0013 (15)	−0.0261 (19)
O3	0.053 (2)	0.0349 (18)	0.0454 (19)	0.0047 (15)	−0.0045 (15)	−0.0064 (15)
O4	0.026 (2)	0.029 (2)	0.031 (2)	0.000	0.0003 (16)	0.000
O5	0.025 (2)	0.100 (4)	0.032 (2)	0.000	0.0016 (19)	0.000
O6	0.058 (2)	0.0403 (19)	0.0434 (19)	−0.0019 (16)	0.0097 (16)	0.0080 (15)
O7	0.0285 (17)	0.078 (3)	0.053 (2)	0.0039 (17)	0.0007 (15)	−0.0328 (19)
O8	0.065 (4)	0.169 (7)	0.037 (3)	0.000	0.012 (3)	0.000
C1	0.030 (2)	0.037 (3)	0.038 (3)	0.009 (2)	−0.0053 (19)	−0.011 (2)
C2	0.028 (2)	0.029 (2)	0.032 (2)	0.0044 (18)	−0.0058 (18)	−0.0058 (18)
C3	0.037 (2)	0.037 (2)	0.032 (2)	−0.001 (2)	0.0018 (18)	−0.0065 (19)
C4	0.033 (2)	0.036 (2)	0.038 (2)	−0.0057 (19)	−0.0007 (19)	−0.003 (2)
C5	0.030 (2)	0.028 (2)	0.035 (2)	0.0017 (18)	−0.0063 (18)	−0.0048 (18)
C6	0.038 (3)	0.044 (3)	0.034 (2)	0.002 (2)	0.0021 (19)	−0.012 (2)
C7	0.027 (2)	0.045 (3)	0.041 (3)	−0.001 (2)	0.0030 (19)	−0.012 (2)
C8	0.037 (2)	0.033 (2)	0.032 (2)	0.001 (2)	−0.0043 (19)	−0.0060 (19)

### Geometric parameters (Å, °)

**Table:**

Mn1—O5	2.137 (4)	O7—H7D	0.8500
Mn1—O7	2.161 (3)	O8—H8C	0.8500
Mn1—O7^i^	2.161 (3)	C1—C2	1.506 (6)
Mn1—O6^i^	2.187 (3)	C2—C3	1.381 (6)
Mn1—O6	2.187 (3)	C2—C7	1.384 (6)
Mn1—O4	2.225 (4)	C3—C4	1.390 (6)
O1—C1	1.265 (5)	C3—H3A	0.9300
O2—C1	1.248 (6)	C4—C5	1.375 (6)
O3—C8	1.419 (5)	C4—H4	0.9300
O3—H3	0.8200	C5—C6	1.387 (6)
O4—H4C	0.8500	C5—C8	1.520 (6)
O5—H5C	0.8500	C6—C7	1.388 (6)
O5—H5D	0.8500	C6—H6	0.9300
O6—H6C	0.8500	C7—H7	0.9300
O6—H6D	0.8500	C8—C8^ii^	1.548 (8)
O7—H7C	0.8500	C8—H8	0.9800
			
O5—Mn1—O7	91.37 (12)	O2—C1—C2	117.7 (4)
O5—Mn1—O7^i^	91.37 (12)	O1—C1—C2	118.8 (4)
O7—Mn1—O7^i^	95.3 (2)	C3—C2—C7	118.6 (4)
O5—Mn1—O6^i^	94.52 (13)	C3—C2—C1	121.1 (4)
O7—Mn1—O6^i^	171.61 (14)	C7—C2—C1	120.2 (4)
O7^i^—Mn1—O6^i^	90.57 (14)	C2—C3—C4	120.6 (4)
O5—Mn1—O6	94.52 (13)	C2—C3—H3A	119.7
O7—Mn1—O6	90.57 (14)	C4—C3—H3A	119.7
O7^i^—Mn1—O6	171.61 (14)	C5—C4—C3	120.7 (4)
O6^i^—Mn1—O6	83.01 (19)	C5—C4—H4	119.6
O5—Mn1—O4	178.63 (17)	C3—C4—H4	119.6
O7—Mn1—O4	87.71 (11)	C4—C5—C6	119.0 (4)
O7^i^—Mn1—O4	87.71 (11)	C4—C5—C8	119.9 (4)
O6^i^—Mn1—O4	86.50 (12)	C6—C5—C8	121.1 (4)
O6—Mn1—O4	86.50 (12)	C5—C6—C7	120.1 (4)
C8—O3—H3	109.5	C5—C6—H6	119.9
Mn1—O4—H4C	121.4	C7—C6—H6	119.9
Mn1—O5—H5C	112.2	C2—C7—C6	120.9 (4)
Mn1—O5—H5D	139.5	C2—C7—H7	119.5
H5C—O5—H5D	108.3	C6—C7—H7	119.5
Mn1—O6—H6C	113.5	O3—C8—C5	111.9 (3)
Mn1—O6—H6D	109.4	O3—C8—C8^ii^	105.9 (4)
H6C—O6—H6D	108.4	C5—C8—C8^ii^	111.9 (4)
Mn1—O7—H7C	125.7	O3—C8—H8	109.0
Mn1—O7—H7D	117.3	C5—C8—H8	109.0
H7C—O7—H7D	108.4	C8^ii^—C8—H8	109.0
O2—C1—O1	123.5 (4)		
			
O2—C1—C2—C3	176.5 (4)	C4—C5—C6—C7	0.3 (7)
O1—C1—C2—C3	−3.0 (6)	C8—C5—C6—C7	179.7 (4)
O2—C1—C2—C7	−1.2 (6)	C3—C2—C7—C6	−0.5 (7)
O1—C1—C2—C7	179.3 (4)	C1—C2—C7—C6	177.3 (4)
C7—C2—C3—C4	0.1 (7)	C5—C6—C7—C2	0.3 (7)
C1—C2—C3—C4	−177.6 (4)	C4—C5—C8—O3	−128.0 (4)
C2—C3—C4—C5	0.5 (7)	C6—C5—C8—O3	52.5 (6)
C3—C4—C5—C6	−0.7 (7)	C4—C5—C8—C8^ii^	113.3 (6)
C3—C4—C5—C8	179.9 (4)	C6—C5—C8—C8^ii^	−66.1 (6)

Symmetry codes: (i) *x*, −*y*+3/2, *z*; (ii) −*x*+1, −*y*+1, −*z*+1.

### Hydrogen-bond geometry (Å, °)

**Table:**

*D*—H···*A*	*D*—H	H···*A*	*D*···*A*	*D*—H···*A*
O3—H3···O1^iii^	0.82	2.02	2.830 (5)	172
O4—H4C···O1^iv^	0.85	1.86	2.712 (4)	177
O5—H5C···O4^v^	0.85	1.93	2.777 (6)	175
O5—H5D···O8^v^	0.85	1.88	2.728 (7)	175
O6—H6C···O3^ii^	0.85	1.99	2.840 (5)	178
O6—H6D···O8	0.85	2.19	3.040 (6)	178
O7—H7C···O1^vi^	0.85	1.95	2.799 (5)	180
O7—H7D···O2^iv^	0.85	1.82	2.673 (4)	180
O8—H8C···O2^vii^	0.85	1.92	2.767 (5)	172

Symmetry codes: (iii) −*x*+2, −*y*+1, −*z*+2; (iv) *x*−1, *y*, *z*−1; (v) *x*+1, *y*, *z*; (ii) −*x*+1, −*y*+1, −*z*+1; (vi) *x*, *y*, *z*−1; (vii) *x*−1, *y*, *z*.
